# Inflammatory abrasion of hematopoietic stem cells: a candidate clue for the post-CAR-T hematotoxicity?

**DOI:** 10.3389/fimmu.2023.1141779

**Published:** 2023-05-08

**Authors:** Ting Sun, Dengju Li, Liang Huang, Xiaojian Zhu

**Affiliations:** Department of Hematology, Tongji Hospital, Tongji Medical College, Huazhong University of Science and Technology, Wuhan, China

**Keywords:** cytopenia, hematotoxicity, inflammation, CAR-T, hematopoietic stem cell

## Abstract

Chimeric antigen receptor T-cell (CAR-T) therapy has shown remarkable effects in treating various hematological malignancies. However, hematotoxicity, specifically neutropenia, thrombocytopenia, and anemia, poses a serious threat to patient prognosis and remains a less focused adverse effect of CAR-T therapy. The mechanism underlying lasting or recurring late-phase hematotoxicity, long after the influence of lymphodepletion therapy and cytokine release syndrome (CRS), remains elusive. In this review, we summarize the current clinical studies on CAR-T late hematotoxicity to clarify its definition, incidence, characteristics, risk factors, and interventions. Owing to the effectiveness of transfusing hematopoietic stem cells (HSCs) in rescuing severe CAR-T late hematotoxicity and the unignorable role of inflammation in CAR-T therapy, this review also discusses possible mechanisms of the harmful influence of inflammation on HSCs, including inflammatory abrasion of the number and the function of HSCs. We also discuss chronic and acute inflammation. Cytokines, cellular immunity, and niche factors likely to be disturbed in CAR-T therapy are highlighted factors with possible contributions to post-CAR-T hematotoxicity.

## Introduction

Chimeric antigen receptor T-cell (CAR-T) therapy is a type of adoptive T-cell immunotherapy (1), in which T cells are genetically modified to express a CAR consisting of a specific antigen recognition domain from a B-cell receptor and essential signaling elements for T cells (1). The antigen recognition domain is encoded by single-chain variable fragments (scFv) and can be substituted to target various kinds of cells. The signaling elements are composed of a co-stimulation domain (two co-stimulation domains for the third generation of CAR) and a signal transduction domain (1, 2). The fourth generation of CAR also contains a cytokine secretion domain (3). Such genetic modification capacitates CAR-T cells with vigorous non-major-histocompatibility-complex (MHC)-restricted cytotoxicity (1). Cytokines released during the eradication of target cells also activate neighboring immunocytes, which can exert a synergistic therapeutic effect (1). Subsequently, part of the CAR-T cells may enter the memory pool, circulating in the body for a long time and supervising the primary disease (1).

CAR-T therapy has achieved encouraging success in relapsed/refractory (R/R) hematological malignancies (4). In patients with R/R non-Hodgkin lymphoma (NHL), the long-term follow-up of Axicabtagene ciloleucel, an anti-CD19 CAR-T therapy, reported a median overall survival (OS) of 25.8 months [95% confidence interval (CI), 12.8–NE] (4), while the median OS was only 6.3 months (95% CI, 5.9–7.0) in R/R NHL patients (*n* = 636) treated with chemotherapies or autologous stem cell transplantation (5). The application of CAR-T has also extended to solid carcinomas, presenting a challenging and promising prospect (6). However, specific adverse events emerge simultaneously, such as cytokine release syndrome (CRS), immune effector cell-associated neurotoxicity syndrome (ICANS), B-cell aplasia, hypogammaglobulinemia, and hematotoxicity (i.e., cytopenia) (7, 8). The most highlighted hematotoxic events are neutropenia, thrombocytopenia, and anemia (9, 10). Furthermore, leukopenia and lymphocytopenia are also the manifestations of cytopenia.

CAR-T late hematotoxicity refers to cytopenia of single or multiple lineages in recurrence, persistent myelosuppression (11–17), or occasionally reported delayed onset (12, 14). However, there is still a lack of consensus on the exact time to determine a late event in cytopenia. Cytopenia that occurs immediately after the infusion may recover gradually during residency. However, delayed or recurrent cytopenia may occur (12, 15) during the outpatient follow-up and might miss timely intervention. Moreover, persistent aplasia could prolong the time of hospitalization, adding to the medical expenses and the difficulty of clinical management (18). Severe cytopenia casts a shadow on the survival of the patients, raising the risk of severe infection, lethal bleeding, and extreme fatigue (10). Therefore, CAR-T hematotoxicity, specifically late events, should be studied thoroughly.

Hematopoietic stem cells (HSCs) lie at the top hierarchy of the hematopoietic output (19). Several clinical studies have reported the success of transfusing HSCs, also called hematopoietic stem cell boosting (HSCB), in rescuing severe and prolonged hematotoxicity (20–22). The importance of HSCs in the pathology of CAR-T hematotoxicity has been highlighted, although the role of differentiated and mature hematocytes could not be excluded. Moreover, patients are persistently challenged by inflammation owing to the previous multiple lines of chemotherapies or radiotherapies, CRS following CAR-T therapy, infections, and other pro-inflammatory events. Therefore, inflammation is likely a vital factor influencing hematopoietic recovery after CAR-T infusion.

In this review, we aim to discuss the possible mechanisms underlying CAR-T late hematotoxicity considering the negative impact of inflammation on the hematopoietic system, especially on HSCs. We aim to summarize current studies on the features of CAR-T late hematotoxicity, potential influencing factors, clinical management, and pathogenesis. Moreover, the crucial role of HSCs and inflammation as well as the negative influence of inflammation on HSCs will also be reviewed to provide information on pathogenic mechanisms.

## Clinical studies on CAR-T late hematotoxicity

### Definition and characteristics

Diagnosis and evaluation of CAR-T hematotoxicity are based on the Common Terminology Criteria for Adverse Events (CTCAE) (11–17). Despite this consensus, the definition of CAR-T late hematotoxicity, summarized in **Table 1**, varies in studies. One divergence was the severity of cytopenia. Most studies concentrated on grade 3–4 cytopenia, while less attention was given to grade 1–2 cytopenia, because more severe cytopenia might correlate with a higher incidence of infection (18, 23), lethal bleeding, and a worse prognosis (10, 18), thus requiring more intensive investigation. However, milder cytopenia should not be neglected because the main goal is normalizing hematopoietic recovery. Another divergence is the time point to define a late event, which went from 21 to 90 days post-infusion (11–17) (**Table 1**). Based on these existing reports, a standard time point for late events remains unclear.

**Table 1:** Definition of CAR-T late hematotoxicity and severity of cytopenia of concern in different studies.

Reference	Disease	CAR-T Target	Definition	Patients (*n*)	Severity of concern graded by CTCAE			Incidence			Risk factors	
					Neutropenia	Thrombocytopenia	Anemia	Neutropenia	Thrombocytopenia	Anemia	Baseline	Post infusion
**Cordeiro t al. (2020)** (11)	NHL CLL ALL	CD19	Cytopenia requiring transfusion or growth factor support after day 90 post infusion.	19	3–4	4	4	16%	11%	11%	NR	NR
**Fried et al. (2019)** (12)	NHL ALL	CD19	ANC < 1.5 × 10^9^/L, and PLT < 150 × 10^9^/L, after day 21 post infusion.	29	2	1	NR	76%	76%	NR	Prior HSCT	CRS SDF-1
			ANC < 0.5 × 10^9^/L, PLT < 50 × 10^9^/L and anemia requiring transfusing were classified as severe.		4	3–4	4	34%	21%	17%		
**Brudno et al. (2022)** (13)	NHL MM	CD19 BCMA	ANC < 1 × 10^9^/L, PLT < 50 × 10^9^/L, and Hb < 80 g/L, for recurrence or prolonging cytopenia after day 30 post-infusion.	35^a^	3–4	3–4	3–4	37%	11%	6%	Baseline anemia and neutropenia	CAR T-cell persistence in BM aspirate
				13^b^				15%	23%	15%		
**Wang et al. (2021)** (14)	ALL	CD19 CD19/CD22	ANC < 0.5 × 10^9^/L, Hb < 60 g/L, PLT < 20 × 10^9^/L, on day 28 post infusion.	76	4	4	4	25%^c^			Severe baseline cytopenia, bone marrow tumor burden, LDH.	CRS, CRP, ferritin, D-dimer, IFN-γ, IL-10, usage of tocilizumab/steroids
**Rejeski et al. (2022)** (15)	B-cell lymphoma	CD19	ANC < 1 × 10^9^/L after day 21 post infusion.	235	3–4	NR	NR	64%	NR	NR	Baseline cytopenia, CRP, ferritin	—
**Nagle et al. (2021)** (16)	DLBCL	CD19	ANC < 1 × 10^9^/L, and PLT < 50 × 10^9^/L, after day 30 post infusion.	31	3–4	3–4	NR	58%	48%	NR	—	CRS, tocilizumab/steroids, peak ferritin, peak CRP
**Strati et al. (2021)** (17)	Large B-cell lymphoma	CD19	Grade 3–4 cytopenia after day 30 post infusion.	31	3–4	3–4	3–4	29%	42%	16%	ECOG = 1, >3 prior therapies, low ALC.	—

^a^ Thirty-five patients were evaluable for recurrent cytopenia.

^b^ Thirteen patients were evaluable for prolonging cytopenia.

^c^ Lineage-specific incidence not reported.

Time point selected by different studies to define a late event is underlined. NHL, non-Hodgkin lymphoma; CLL, chronic lymphocytic leukemia; ALL, acute lymphocytic leukemia; MM, multiple myeloma; DLBCL, diffuse large B-cell lymphoma; ANC, absolute neutrophil count; PLT, platelet count; Hb, hemoglobin concentration; NR, not reported; SDF-1, stromal cell derived factor-1; ECOG, Eastern Cooperation Oncology Group. - means not reported.

**Figure 1:**
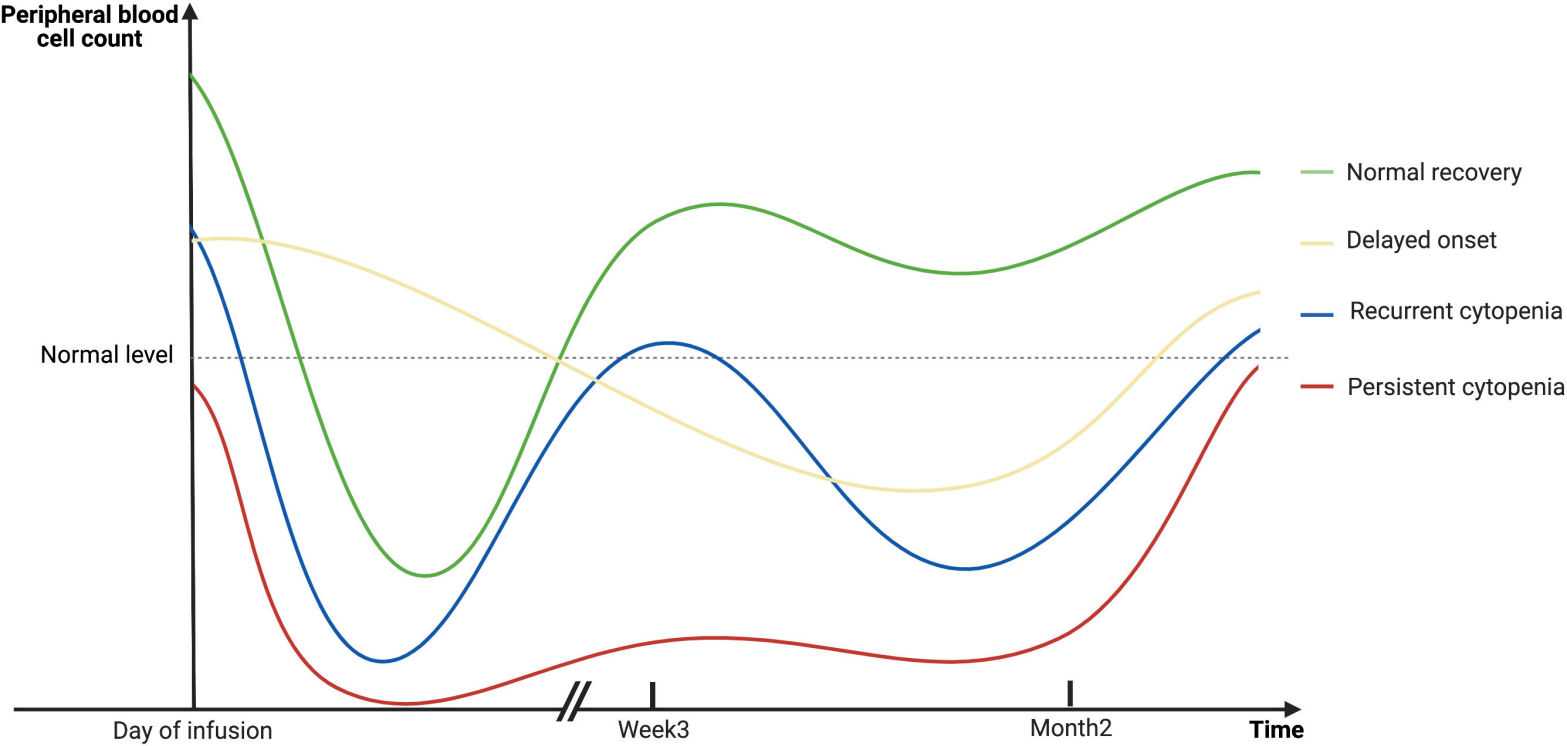
A schematic diagram showing the dynamics of hematopoietic reconstitution in CAR T-cell-treated patients. The early phase of cytopenia may be related to lymphodepletion chemotherapies and CRS, and will gradually recover in the majority of patients within 3 weeks after infusion. However, late-phase events, which are in the form of delayed-onset, recurrent, or persistent cytopenia in the second month or even later, cannot be explained for the same reasons. Created with BioRender.com.

Among patients with B-cell lymphoma and treated with anti-CD19 CAR-T cells, the onset of cytopenia was observed from the day of infusion, or occasionally up to a month post-infusion (12). The median time to onset of neutropenia and thrombocytopenia was 3 days (range, 0–21) and 0 days (range, 0–38), respectively (12). For those patients who developed cytopenia a few days following infusion, previous studies reported a “biphasic” pattern of hematopoietic recovery (12, 15). The first trough of neutrophil count recovered within 3 weeks in 77% of the patients (*n* = 149) (15). However, 52% of the patients will experience a second trough of neutrophil count after a month of infusion (15). Moreover, some patients underwent severe myelosuppression, which could prolong for weeks, months, or even years without recovery and could be resistant to intensive clinical interventions (11, 13, 15, 17, 22, 24). Researchers held the opinion that the early phase of cytopenia might be related to lymphodepletion chemotherapies and CRS (12, 15). However, these factors should have subsided within 3 weeks of infusion (12, 15). Therefore, late-phase events, which are in the form of delayed-onset, recurrent, or persistent cytopenia, might be attributed to unknown mechanisms and require further investigation. **Figure 1** depicts the different dynamics of hematopoietic reconstruction in CAR T-cell-treated patients.

### Incidence and risk factors

The incidence of CAR-T hematotoxicity reported by different clinical trials has been thoroughly reviewed recently (6, 10). Briefly, the overall incidence of severe (grade ≥ 3) neutropenia, thrombocytopenia, and anemia ranges from 29% to 95%, from 15% to 65%, and from 14% to 77%, respectively (6, 10). However, a fewer number of studies distinguished late-phase from early-phase cytopenia. Those studies specifying CAR-T late-phase (>21 days post-infusion) hematotoxicity reported the highest incidence of grade 3–4 neutropenia, thrombocytopenia, and anemia to be 64%, 48%, and 17%, respectively (**Table 1**).

Retrospective studies have analyzed the clinical characteristics to identify associated factors related to CAR-T late hematotoxicity (**Table 1**). These studies were carried out among patients with R/R B-cell malignancies. CD19 was the most common therapeutic target, while other targets like B-cell maturation antigen (BCMA), CD22, and multi-target therapies were less reported. Juvenile patients were included in the analysis (12). However, no specific attention was given to the underage subgroup, and existing reports found no correlation between age and the incidence and severity of hematotoxicity (11–17). For the risk factors, different studies arrived at contradictory conclusions, specifically on the severity of CRS. Several studies reported that increased CRS severity was a predictor for slower recovery from cytopenia in the first month after CAR-T infusion (13, 25, 26). In a study of 83 patients with B-cell malignancies, grade ≥3 CRS was significantly associated with the absence of complete hematological recovery at a month (*p* = 0.002) (25). In another study (*n* = 164), the mean time to neutrophil recovery was 4.2 and 12.8 days in patients with grade 0 and ≥3 CRS, respectively (26). These findings indicated that CRS might be a predictor for the persistence of early-phase cytopenia. Moreover, some reports have stated that late hematotoxicity might be more common in patients with severe CRS grades (12, 14). Higher CRS grade showed a strong correlation with cytopenia > 21 days (*p* = 0.003, 0.018, and 0.04 for late anemia, thrombocytopenia, and anemia, respectively) (12). However, some reports stated otherwise (15, 17, 25, 27). The largest population (*n* = 235), and also the only multi-center analysis of CAR-T hematotoxicity carried out in R/R B-cell lymphoma patients revealed that CRS severity was not a risk factor (15). Aside from CRS, the cytokine profile was also analyzed. However, no specific cytokine was identified as a dependent influencing factor for hematotoxicity (12–14, 25, 26). Therefore, the specific contribution of cytokines to late hematotoxicity is difficult to determine. Other inflammatory indicators like C-reactive protein (CRP) and ferritin were correlated factors (14–16). Aside from inflammation-associated factors, baseline (before lymphodepleting chemotherapies) cytopenia was repeatedly reported as a strong indicator of persistent myelosuppression after CAR-T infusion (13–15, 17). For all 35 patients with NHL or MM available for evaluation, the hemoglobin level was lower among patients who developed delayed cytopenia (*p* = 0.0079) (13). For 76 patients with acute lymphocytic leukemia (ALL), researchers also identified that patients without baseline cytopenia exhibited easier hematologic restoration (*p* = 0.028) (14). These findings indicated that baseline cytopenia was strongly related to CAR-T hematotoxicity in different diseases.

CAR-T HEMATOTOX (HT), the only available predictive model for severe CAR-T hematotoxicity, was proposed based on the analysis of clinical characteristics in adult patients with R/R B-cell lymphomas (15). This evaluation system strengthens the importance of clinical features at baseline, including peripheral counts of neutrophils and platelets, hemoglobin concentration, CRP, and ferritin levels. Patients with lower baseline peripheral counts and higher CRP and ferritin levels will get a higher HT score. They are more likely to develop severe infections, specifically bacterial infections, and are more vulnerable to non-relapsed mortality (23). Although HT successfully predicts the occurrence of severe and prolonged neutropenia lasting longer than 14 days post-infusion (15), the recurrence of severe cytopenia cannot be indicated, nor can it reveal the prognosis of the patients with severe hematotoxicity (28). Therefore, future efforts must be dedicated to optimizing the evaluation model.

### Clinical interventions

Clinical interventions for CAR-T late hematotoxicity are typically supportive and without specific targets. The most common strategies are blood transfusion, growth factor utilization, and HSCB (20–22, 29). Growth factors are widely used interventions for cytopenia for various reasons. However, only recently have their therapeutic effects on CAR-T hematotoxicity management been carefully investigated (29). A study of 197 patients revealed that, while prophylactic administration of granulocyte-colony stimulating factor (G-CSF) could shorten the duration of neutropenia after CAR-T infusion, reducing later recurrence is ineffective (29). Thrombopoietin receptor agonists (TPO-RA) have also been reported in treating prolonged myelosuppression after CAR-T infusion (30–32). In a retrospective study, 11 patients were treated with TPO-RA, a median of 17 days was needed to gain infusion independency, and a median of 46 days was needed to gain a plate recovery of ≥50 × 10^9^/L after the initiation of TPO-RA (31). Another study has reported similar results in six patients with transfusion-dependent cytopenia (32). However, determining the exact benefit of TPO-RA in these retrospective studies is difficult because no comparison has been made between treated and non-treated patients.

Two clinical studies reported the effectiveness of HSCB in relieving CAR-T late hematotoxicity (20, 21). In a study with 12 patients, the median duration of severe neutropenia and thrombocytopenia was 42 days after CAR-T infusion and the cumulative response rate at day 30 after HSC infusion was 82% for neutropenia and 60% for thrombocytopenia (21). In another study with 31 patients, the response rate for neutropenia was 84%, and the responding patients showed higher survival than non-responding patients (20). HSCB was reported to be ineffective in rescuing patients of CAR-T late hematotoxicity during severe infection (20). However, previous research proposed that infusion of HSC could improve the survival rate of sepsis by 50%–60% (33), indicating that HSCB might still be considered an effective therapy for patients with severe infection, but HSCB is limited by its availability. The failure of mobilization is a challenge, specifically for patients with baseline cytopenia. Alternatively, allogenic sources of HSCs can be used occasionally (20, 21); however, allogenic sources may require careful evaluation for safety and efficiency. Hematotoxicity resistance to HSCB is also a challenging issue. In a case report, clinicians used rapamycin (Sirolimus), an mechanistic target of Rapamycin (mTOR) inhibitor, to suppress CAR-T amplification and successfully rescued a heavily treated patient with persistent myelosuppression who had failed HSCB (24).

## Investigations on the pathogenesis of CAR-T late hematotoxicity

Aside from clinical features, a few reports shed light on the pathogenesis of CAR-T late hematotoxicity. A deep sequencing approach to determine the prevalence of clonal hematopoiesis of indeterminate potential (CHIP), which is closely associated with chronic inflammation, revealed that CHIP is not related to the dynamics of hematopoietic recovery (34). In another study, single-cell analysis and serum cytokine analysis revealed a case of aplastic CAR-T hematotoxicity with bone marrow failure featuring oligoclonal T-cell expansion and altered cytokine-related features (35). Inflammation is the current focus of discussion, and further research is urgently needed for the pathogenetic investigations of CAR-T late hematotoxicity.

## Negative influences of inflammation on HSCs

Several reviews have discussed the possible mechanisms of CAR-T hematotoxicity (6, 10). Maintaining HSC homeostasis, the interaction between bone marrow niches, regulation by inflammatory cytokines, disorders of cellular immunity, and others could all play a part in the complex mechanisms (6, 10). However, no clinical disorders referred to by these detailed reviews (6, 10), such as acquired aplastic anemia (AA) and myelodysplastic syndromes (MDS), could fully concur with the characteristics of CAR-T late hematotoxicity. The biphasic or aplastic recovery feature indicates an intermittent or profound reduction of hematopoietic cells and a temporary or prolonged impairment of hematopoietic function. Considering the success of HSCB in relieving CAR-T late hematotoxicity and the crucial role of inflammation during CAR-T therapy, the negative role of inflammation on HSCs can be summarized.

### Reducing the number of HSCs

Homeostatic hematopoietic output is a carefully regulated hierarchy process. In homeostasis, most HSCs are quiescent in the G0 phase (36). While long-term HSCs (LT-HSCs) are responsible for lifelong persistent hematopoiesis, multipotent progenitors (MPPs) dominate the homeostatic hematopoietic output. MPP1 is a cluster of metabolically active HSCs, also identified as short-term HSCs (ST-HSCs) (19); MPP2–4 are subsets of lineage-biased MPPs with reduced self-renewal potency, of which the most abundant MPP4 generates primarily myeloid and lymphoid output (37). MPPs may serve as the hematopoietic buffer, rapidly adapting to stimulations without uncontrolled activation of HSCs, which may be detrimental (37).

Maintaining the balance between quiescence and proliferation is essential for a long-term stabilized stem cell pool (38). However, this homeostasis can be disrupted when confronted with challenges such as inflammation. Intrinsic or extrinsic cell death mechanisms are also important factors contributing to diminishing the number of hematopoietic cells.

#### Disturbed quiescence of HSCs

Quiescent HSCs harbor the most robust self-renewal capacity, and they are indispensable for a persistent stable hematopoietic output (39, 40). Quiescent HSCs exhibit better multi-lineage repopulation ability in long-term transplantation experiments (41). The conferring of dormant HSCs into an active cell cycle is not a fully reversible process, generating daughter phenotypic HSCs, which may not be as potent even after the return to dormancy (42, 43). Moreover, consistent activation and frequent division are detrimental to self-renewal and may eventually result in the depletion of the stem cell pool (44, 45).

The risk of chronic inflammation in HSCs has been well-established (45–48). *In vivo* chronic stimulation of lipopolysaccharide (LPS), pI:pC, or different cytokines, such as interferons (IFNs), transforming growth factors (TNFs), and IL-1 (48–51), induces HSC depletion *via* interruption of quiescence, promoting proliferation and differentiation at the expense of self-renewal. The effects can be either directly mediated by Toll-like receptors (TLRs) (52) or cytokine receptors on HSCs (53) or indirectly mediated *via* interfering with bone marrow niches (54). Chronic inflammation due to multiple lines of therapies and tumor-bearing status may already reduce the number and potency of HSCs before CAR-T treatment. Some patients certainly developed cytopenia before the routine lymphodepleting chemotherapy, which is a predictive factor of severe and prolonged cytopenia after CAR-T infusion (13–15, 17). However, chronic inflammation is constantly related to myeloid-biased hematopoietic output (55), which is commonly observed in MDS but seldom reported in CAR-T hematotoxicity. CHIP has not been proven to contribute to CAR-T hematotoxicity (34).

Acute inflammation also exerts influences on HSCs, although whether this effect is prolonged varies between investigations. A previous study showed that acute inflammation was not likely to considerably influence HSC potency in the long term (56). However, later investigations indicated that the impairment was prolonged or persistent after acute inflammation (41, 43). Peripheral virus infection activated HSCs by inflammatory cytokines and chemokines (43). The bone marrow of murine cytomegalovirus (MCMV)-infected mice was extracted at 4 days, 21 days, and 4 months post-infection. Competitive and secondary transplantation experiments revealed a significantly impaired function both in HSCs harvested 4 days post-infection during the acute phase and in HSCs harvested 21 days post-infection after returning to phenotypic quiescence (43). However, such impairment was not observed in the bone marrow harvested 4 months post-infection, indicating a long-term, but not infinite, impairment (43). In a more recent investigation (41), wild-type C57BL/6J mice were injected with blocks of pI:pC to mimic rounds of discrete acute inflammation in moderate intensity during virus infection. LT-HSCs demonstrated more rapid differentiation kinetics and faster exit form quiescence than their phosphate buffer saline (PBS)-treated counterparts, along with compromised overall *in vitro* proliferative potential (41). HSCs from mice treated with three blocks of pI:pC (i.e., eight individual injections spread over 8 weeks for each block) showed a significantly reduced functional potency than their age-matched counterparts even after a 12-month recovery (41). Further investigation of the Scl-tTA; H2BGFP mouse model revealed that H2B-GFP-retaining undivided HSCs, which remained quiescent throughout the challenges, maintained better functional potency than the divided subset, and the shrink of the dormant HSC pool was responsible for a persistently impaired hematopoietic output (41). Acute inflammation such as CRS or infection is common after CAR-T therapy. Therefore, acute inflammation may contribute at least partially to temporary or prolonged cytopenia in this scenario. However, clinical investigations have contradictory conclusions as to whether the grade of CRS has associated with CAR-T late hematotoxicity. Given the distinctive cytokine profile of CRS from viral infection (43, 57), CRS needs to be verified whether the profound effects of acute inflammation on LT-HSCs observed in viral infection are also applicable to CAR-T therapy.

**Figure 2:**
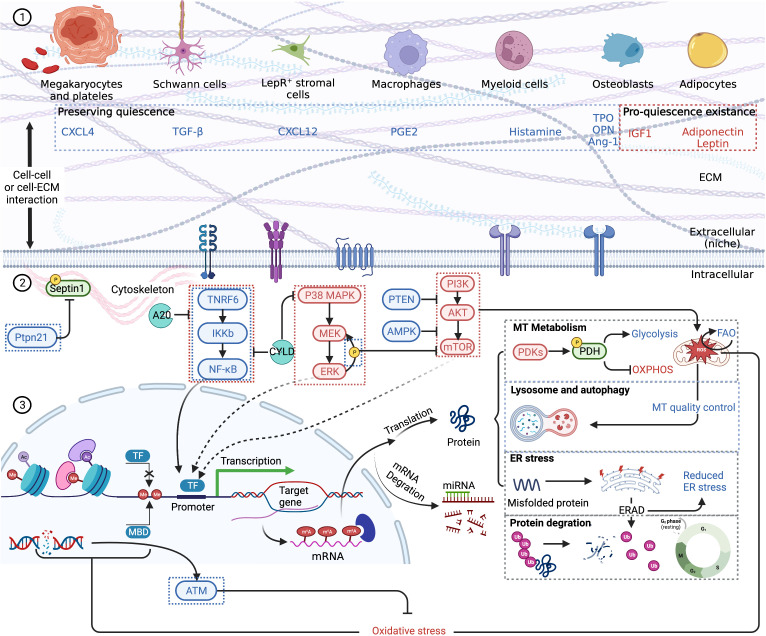
A schematic summary of HSC quiescence regulation based on previous reviews (58, 59). Blue font and boxes label the factors maintaining quiescence, while red font and boxes label the factors promoting quiescence existence. (1) HSC extrinsic regulation. Niche cells and respective cytokines are pictured. HSCs receive stimulation *via* membrane receptors. Cell–cell interaction and cell–ECM interaction are also vital to quiescence retention. (2) HSC intrinsic regulation. NF-kB pathway, MAPK pathway, and mTOR pathway are the main signal pathways regulating HSC quiescence. Quiescent HSCs mainly depend on glycolysis for energy, and the self-renewal and maintenance of stem-cell pool rely on FAO. Lysosome and autophagy are important to clear excessively active mitochondria. Epigenetic changes, including histone and DNA modification, which influence gene transcription, are also the consequences of oxidative stress. After transcription, m6A modifications are significant epigenetic factors regulating HSC quiescence. Misfolded or unfolded protein depends on ERAD to degrade *via* ubiquitination. Other proteins including cell cycle proteins are also eliminated by ubiquitination, thus regulating cell dormancy and cycle activation. CXCL, CXC motif chemokine ligand; TGF, transforming growth factor; PGE, prostaglandin E; TPO, thrombopoietin; OPN, osteopontin; IGF, insulin-like growth factor; ECM, extracellular matrix; MT, mitochondrion; PDK, PDH kinases; PDH, pyruvate dehydrogenase; OXPHOS, oxidative phosphorylation; ER, endoplasmic reticulum; TF, transcription factor; MBD, methyl binding domain protein; ATM, ataxia telangiectasia mutated kinase. Created with BioRender.com.

Previous studies show that chronic and acute inflammation can activate dormant HSCs into proliferation at the expense of self-renewal, though activation of only a small clutch of HSCs is not likely to provide a grievous blow to the hematopoietic system (41, 56). A complicated network of intrinsic and extrinsic factors, such as cell cycle regulators, transcription factors, epigenetic factors, niche factors, and metabolism regulators, is involved in maintaining the quiescence of HSCs and has been thoroughly reviewed previously (**Figure 2**) (58, 59). Pathologic gene mutations are common in patients with hematological malignancies. For instance, P53, a commonly tested gene mutation in clinical practice, is an important transcription factor regulating HSC quiescence (58). Whether P53, along with other gene mutations, is associated with the incidence of CAR-T late hematotoxicity remains unknown. Moreover, epigenetic factors should be carefully considered. Inflammation has been proven to exert a long-lasting epigenetic effect on LT-HSCs, in a way designated as “trained immunity” (60) or leading to accelerated senescence (41). The contribution of epigenetic alternations to late hematotoxicity remains unknown. Niche factors, which are highly likely to be disrupted, should also be emphasized. For example, CD150 high bone marrow regulatory T cells (Tregs) maintain HSC quiescence *via* adenosine (61). However, Tregs are reduced during CAR-T therapy due to lymphodepleting chemotherapy, although whether niche-resident Tregs are also depleted remains unidentified. Hence, an essential factor in maintaining HSC dormancy would be diminished. Furthermore, inflammation is strongly associated with increased reactive oxygen species (ROS) and altered HSC metabolism, regulating glycolysis, oxidative phosphorylation (OXPHOS), and fatty acid oxidation (FAO), eventually influencing fate decisions (45, 62).

#### Cell death mechanisms of HSCs

Under inflammatory challenges, different forms of cell death take part in reducing the number of hematopoietic cells, such as apoptosis, necroptosis, and pyroptosis (47). Additionally, direct attack by immunity cells also leads to cell death. While dormant HSCs tend to be more resistant to harmful factors than differentiated hematocytes (41, 63, 64), HSCs can be conferred to death under certain circumstances.

In inflammation, some cytokines like IFNs and TNFs are proapoptotic (54). HSCs were primed to apoptosis under chronic exposure to type I IFNs. Once forced into the cell cycle, such as cell culture, transplantation, and myeloid-ablative treatment, the p53-dependent proapoptotic program was quickly activated, leading to HSC depletion and bone marrow failure (64). An earlier investigation revealed that IFN-γ treatment upregulated FAS along with other proapoptotic genes, sensitizing Lin-Sca-1+c-Kit- cells (LSKs) to apoptosis both *in vitro* and *in vivo* (65). In addition, LSKs consist of different cell subsets with heterogeneous vulnerability to the same treatment (66, 67); hence, earlier reports on these poorly purified populations should be judged carefully. For example, HSCs and MPP2/3 were resistant to the cytotoxicity of TNFα, while MPP4, granulocyte/macrophage progenitors (GMPs), and other committed and mature cells were susceptible to apoptosis in a dose-dependent manner, indicating that the neutrophil lineage may be more vulnerable (67). Moreover, TNFα initiates necroptosis in a receptor-interacting serine/threonine kinase 1 (RIPK1)-dependent manner (68).

Necroptosis and apoptosis share a common molecular pathway. Apoptosis can transform into necroptosis when suppressed (69, 70). Apoptosis-defect mice with Bax, Bak, and Bid triple knockout showed massive necroptosis in myeloid progenitors, mediated by abnormally raised RIPK1 expression (68). Increased cytokine levels and excessive stem cell proliferation were observed and eventually led to stem cell exhaustion and bone marrow failure (BMF) (68). Knockdown of one RIPK1 allele to normalize RIPK1 expression to wild-type level or inhibition of TNFα signal effectively increased myeloid progenitors and ameliorated cytopenia (68). However, a deficiency of RIPK1 resulted in the overactivation of RIPK3 and MLKL, leading to necroptosis and hematopoietic stem/progenitor cell (HSPC) depletion, indicating that a normal level of RIPK1 is necessary for HSPC survival from necroptosis (71). TNFα is a commonly focused cytokine in necroptosis, inducing apoptosis or necroptosis to lineage-committed progenitor and mature cells in a dose-dependent manner (67). However, TNFα mediated the upregulation of the p65/NF-κB-cIAP2 pro-survival pathway in HSCs, protecting them from necroptosis (67). Nevertheless, with the attenuation of the TNFα signal, this pro-survival pathway would quickly get inactivated, creating a vulnerable time window to necroptosis in activated HSCs, which leads to the contraction of the HSC pool and a transient but significant hematopoietic impairment (67). Only after the return to dormancy is this vulnerability removed (67).

Pyroptosis is another vital cell death mechanism. Similar to necroptosis, pyroptosis leads to the production of pro-inflammatory substrates (72). Viral infection and chemotherapy induce massive hematopoietic progenitor cell death *via* NACHT leucine-rich-repeat protein 1 (NLRP1)-mediated pyroptosis (73). Chronic TGF-β exposure followed by pI:pC acute inflammatory stimulation induced prolonged cytopenia. Consistent upregulation of Caspase1, a key molecule in pyroptosis, was observed in HSCs; however, this research did not further confirm pyroptosis to be the exact reason for BMF (64).

**Figure 3:**
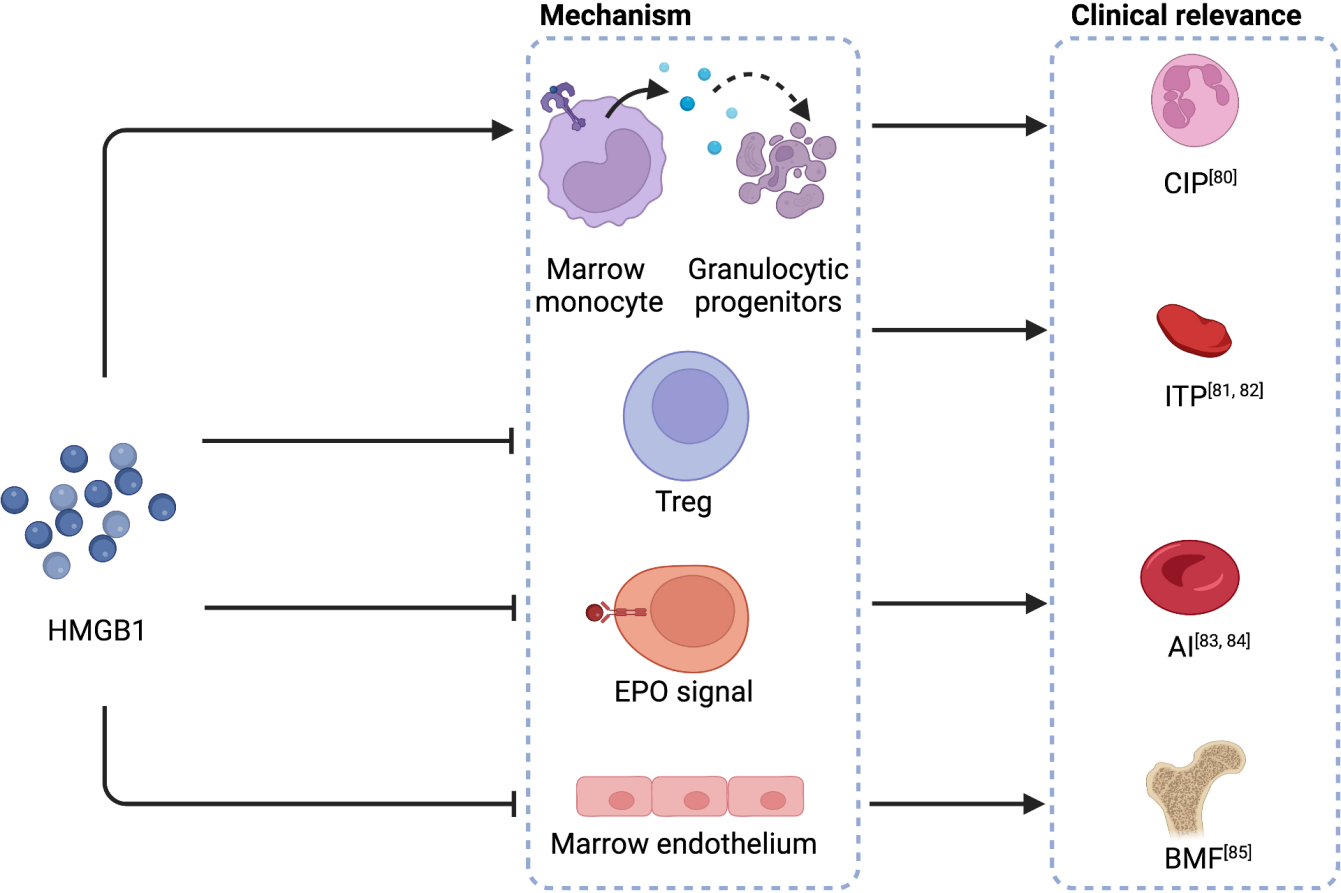
HMGB1 in the mechanisms of cytopenia diseases. HMGB1 causes the apoptosis of granulocytic progenitors, interferes with Tregs, disrupts the regular EPO signal, and impairs bone marrow endothelium, leading to CIP, ITP, AI, and BMF, respectively. HMGB1, high mobility group protein B1; CIP, chronic idiopathic neutropenia; Treg, regulatory T cells; ITP, immune thrombocytopenia; anemia of inflammation, AI; anemia of inflammation; EPO, erythropoietin; BMF, bone marrow failure. Created with BioRender.com.

Necroptosis and pyroptosis lead to the production of damage-associated molecular patterns (DAMPs) (74, 75). As reported in sepsis, DAMPs like high mobility group protein B1 (HMGB1) and mitochondrial DNA (mtDNA) are potent inducers of type I interferons, which negatively regulate emergency hematopoiesis (72). HMGB1 is a late-phase inflammatory substrate secreted by activated megakaryocytes in sepsis (76). While targeting early-phase cytokines gained only limited therapeutic effects, the administration of HMGB1 antagonists significantly improved the survival of systemic inflammatory response syndrome (SIRS) in rodents (77–79). HMGB1 has also been reported to be the mechanism of various cytopenia diseases, even though not directed on HSCs, such as chronic idiopathic neutropenia (CIP) (80), immune thrombocytopenia (ITP) (81, 82), anemia of inflammation (AI) (83, 84), and BMF (85) (**Figure 3**). Pyroptosis is critical for CAR-T assassination of target cells and is proven to be the mechanism of CRS (86). Because one of the dominant HMGB1 receptors, TLR4 (79), is expressed in HSCs (42), HMGB1 may exert a direct impact on HSCs. Nevertheless, whether this impact contributes to CAR-T late hematotoxicity remains unclear.

Besides the influence of cytokines, cellular immunity is also involved in reducing the HSC pool. Cytotoxic T cells attack HSPCs and result in aplastic aplasia (87). In this process, IFN-γ is involved in the recruitment of T cells and the enhancement of the vulnerability of HSPCs to CD8+-T-cell cytotoxicity and mediates the cytotoxic effect (65). Natural killer (NK) cells also participate in the direct attack of HSPCs. NK cells are reported to reduce the number of HSPCs with genotoxic stress by exerting cytotoxicity through the interaction between natural killer group 2 member D (NKG2D) and NKG2D ligand. Inhibition of this interaction could improve cytopenia *in vivo* (88). In normal conditions, HSCs are protected from the attack of cellular immunity. Bone marrow-resident Tregs participate in maintaining an inhibitory immune microenvironment (89, 90). If these Tregs are affected in CAR-T therapy, immune-inhibitive protection would be disrupted. Additionally, CD47 is a “do not eat me” signal expressed on HSCs, while the mobilization of HSCs into the periphery will downregulate its expression (89). DAMPs, which are abundantly generated in CRS (75), were reported to be a powerful mobilization factor (91). SDF-1 level, a cytokine essential for HSC retention in the bone marrow and B-cell development, has been reported to be correlated with late neutropenia, though another report later challenged this conclusion (12, 13). Whether these factors contribute to the reduction of the HSC pool by increasing the vulnerability to cellular immunity attacks is unknown.

### Impairing the function of HSCs

Cytokines are fundamental in regulating hematopoietic output (92), and serum cytokine level has been repeatedly analyzed in clinical reports on CAR-T late hematotoxicity (12–14, 25, 26). Among the diversity of cytokines, including the IL-1 family, hematopoietin (class I cytokine) family, IFN (class II cytokine) family, growth factors, TNF family, IL-17 family, and chemokines (25), some have been identified as relevant to hematopoietic recovery after CAR-T infusion, even though contradictions exist between studies. These cytokines include IL-6, IL-10, IFN-γ, TGF-β1, and SDF-1 (12–14, 25, 26). Higher serum concentrations of IL-6 (26), IL-10, and IFN-γ (14) were associated with lower blood cell counts, while a higher concentration of TGF-β1 (26) and SDF-1 was related to higher blood cell counts (12). IL-6 is a cytokine promoting neutrophil production in emergency hematopoiesis (**Table 2**) (53, 92, 105–107). IL-6 was not included in **Table 2** because it mainly regulates progenitor cells, although the peak level of IL-6 is negatively related to hematopoietic recovery. Moreover, the IL-6 receptor is lowly expressed in HSCs (108). Therefore, even chronically exposing LT-HSCs to IL-6 will not bring apparent functional deficiency (105).

**Table 2:** Cytokines reported to be associated with CAR-T late hematotoxicity and their direct impact on HSCs.

Cytokine	Influences on HSCs	Mechanism	References
**IL-10**	Promoting HSC self-renewal under stressful environment.		(93, 94)
**IFN-γ**	Promoting myeloid differentiation in acute infection;	Upregulating C/EBPβ *via* interaction with Ifngr1	(95)
	Hindering quiescence and promoting excessive terminal differentiation of HSCs in chronic infection;	Disrupting the normally close interaction between HSCs and CXCL12-abundant reticular cells Activating Batf2	(44, 48, 96)
	Navigating HSCs back to quiescence in chronic exposure, but still decreasing their engraftment ability. Inducing HSC cell death upon cell cycle entry.	Might involve Irf2 and Irgm1	(37, 64)
**SDF-1/CXCL-12**	Promoting bone marrow retention, repopulation, and quiescence of HSCs;	Pairing with its receptor CXCR4 and activating multiple signal pathways, e.g., MAPK, PI3K, or PLC	(97, 98)
	Protecting HSCs from depletion in replication stress.	Promoting bone marrow remodeling	
**TGF-β**	Dose-dependent, with low dose stimulating HSC proliferation, and high dose inhibitory;	Activating of MAPK pathway	(99, 100)
	Chronic exposure alters the response of HSCs to acute inflammation, interfering with the ending of inflammation reaction and leading to BMF;	Increasing mitochondrial content, ROS and Caspase-1 activity	(63)
	Impairing HSC function after transplantation;	Upregulating p-Smad2 and pp38^MAPK^	(101)
	Other influences: HSC quiescence, homing, survival and aging.		(102–104)

IL, interleukin, NR, not reported; IFN, interferon; C/EBPβ, CCAAT-enhancer-binding proteins β; CXCL12, C-X-C motif ligand 12; Batf2, basic leucine zipper ATF-like transcription factor 2; Irf2, interferon regulatory factor 2; Irgm1, immunity-related GTPase family M 1; SDF-1, stromal cell derived factor-1; CXCR4, C-X-C receptor 4; MAPK, mitogen-activated protein kinase; PI3K, phosphatidylinositol 3 kinase; PLC, phospholipase C; TGF-β, transforming growth factor-β; BMF, bone marrow failure; ROS, reactive oxygen species; small mothers against decapentaplegic homolog 2.

Cytokines exert a complicated influence on hematopoietic output; however, the actual effects of cytokines tend to be dose-dependent and time-dependent. The dose and acute or chronic exposure may bring opposite influences (53, 54, 109), which has been observed in IL-1 (45, 110–112), TNFα (109), IFNs, and TGF-β (**Table 2**). Besides the direct influence on HSCs, specific cytokines can also interfere with the normal signals controlling hematopoietic output downstream of stem cells. IFN-γ binds to TPO and inhibits hematopoiesis by interfering with the interaction between TPO and its receptor c-MPL as proven in AA (113, 114). In AI, HMGB1 disrupts the signal transduction of erythropoietin (EPO), causing prolonged anemia after sepsis recovery (84, 115). However, as previously shown, the bone marrow cytokine profile might be different from the peripheral (89). Therefore, identifying the bone marrow cytokine profile might be the most accurate and helpful method to determine the critical influencing factors on HSCs after CAR-T therapy.

Some cytokines such as IL-1 (116), IL-6 (54), and TNFα (117, 118) induce inflammation of HSCs (119). The function of HSC is also significantly altered with senescence, even though they still harbor the same stem cell phenotype (117). For one thing, senescent HSCs show diminished hematopoietic potency, myeloid-biased differentiation, and clonal hematopoiesis (117). For another thing, the inflammation-associated signal, like the NF-kB pathway, is upregulated in senescent HSCs, raising their sensitivity to inflammatory challenges (116, 117, 120). However, clinical investigations have shown that older age is not associated with CAR-T late hematotoxicity (11–17). Nevertheless, the biological age of the patient may not reflect the actual age of HSCs. The methyl clock analysis has proved that repeated inflammatory challenges will cause premature aging of LT-HSCs (121). Multiple lines of therapies before CAR-T, which raise inflammatory challenges in bone marrow (122), may be risk factors for the accelerated aging of HSCs. Epigenetic analysis to determine the actual age of HSCs may help clarify the role of senescence in CAR-T late hematotoxicity. mTOR is reported to be vital in regulating the function of senescent HSCs (123). The mTOR signaling pathway is essential for the function of HSCs, by regulating important activities like autophagy, metabolism, and the transformation between quiescence and expansion (124, 125). Rapamycin, an mTOR inhibitor reported to be effective in relieving prolonged CAR-T late hematotoxicity, is an intervention to improve the function of senescent HSCs (123, 125). This mTOR inhibitor has also been proven effective in preserving HSPCs and relieving BMF in mouse models (47). Moreover, apart from cell-intrinsic regulation, the mTOR pathway also regulates the function of bone marrow niches, which provide vital support for normal hematopoiesis. mTORC1 activation enhances the production of IL-19 by osteocytes, and IL-19 has been proven to be a robust stimulation of granulopoieses, relieving chemotherapy- and irradiation-induced neutropenia even more effectively than G-CSF (126).

Niche dysfunction is also harmful to HSCs. Endothelial cells with chronically activated mitogen-activated protein kinase (MAPK) signals are reported to cause HSCs to lose their engraftment ability (127). The impairment of endothelial cells by IFN-γ-induced HMGB1 drives myelosuppression (85). Stromal cells with oxidative stress lead to DNA damage in HSCs (128). Among niche factors promoting hematopoiesis, bone marrow T cells are essential for hematopoietic recovery after stressful challenges. Aside from the function of Tregs, which has already been mentioned earlier in this review, bone marrow-resident group 2 innate lymphoid cells (ILC2) have also been proven vital for hematopoietic recovery as an abundant source of granulocyte-macrophage colony-stimulating factor (GM-CSF) (122). B-cell progenitors produce IL-33, inducing ICL2s to secrete GM-CSF *via* the activation of MyD88, thus promoting neutrophil recovery and bone marrow engraftment (122). ICL2s are also activated by mesenchymal-derived prostaglandin D2 (PGD2) through the interaction with the receptor CRTH2 (129). Activated ICL2s secrete IL-5 and IL-13, and IL-5 further promotes the expansion of HSPCs *via* the stimulation of CD4+CD25+IL5Rα+ Tregs (129). These observations strengthen the importance of niche factors, especially niche-resident T cells in hematopoiesis after stressful challenges. Other niche-resident cells also provide significant cytokines influencing HSC function, such as IL-1β-secreting dendritic cells (DCs) (130) and myeloid cells (116), IL-6-secreting CXCL12-abundant reticular cells (105), IL-19-secreting osteocytes (126), and CXC motif chemokine ligand (CXCL)-12 secreting stromal cells (97, 131).

## Conclusion and future considerations

This review summarizes CAR-T late hematotoxicity, including definition, incidence, clinical characteristics, risk factors, and interventions. Current clinical investigations on CAR-T late hematotoxicity highlight the following: (1) The chosen time point for late events is arbitrary and inconsistent between studies. Determining the exact time point for the late event immediately after CAR-T infusion, based on the duration of earlier cytopenia, may help answer this issue. (2) Research attention is being paid to severe cytopenia of grade 3–4, while less is devoted to cytopenia in grade 1–2. If the normalization of hematopoietic recovery is considered a standard, the challenge of CAR-T late hematotoxicity should be more severe. (3) Baseline cytopenia is a relatively definite indicator of severe late hematotoxicity. Therefore, to eliminate the influence of CAR-T-associated factors, analyses should focus on patients without baseline cytopenia. (4) While HSCB exhibits positive therapeutic effects, the availability of this treatment is limited. Interventions are mainly supportive without definite targets. Moreover, HSCB is mainly for aplastic CAR-T late hematotoxicity, and whether recurrent cytopenia requires positive interventions is unknown.

Owing to the limited research available, the exact pathogenesis is difficult to discuss. Based on the therapeutic effect of HSCB and the influence of inflammation in CAR-T therapy (20–22), we reviewed the negative impact of inflammation on HSCs. HSCB directly supplements the number of HSCs and improves hematopoiesis in the majority of patients, indicating that the loss of HSCs due to undetermined factors contributes to the incidence of CAR-T late hematotoxicity. However, a small proportion of patients were unresponsive to HSCB and showed poor prognosis, indicating that the function of HSCs is crucial. Therefore, we divided the discussion of the negative influence of inflammation on HSCs into the depletion of stem cell number and the impairment of hematopoietic function. Nevertheless, one limitation is that we have not emphasized the more differentiated and mature hematocytes as they have been summarized previously (10) and no clinical practice suggests that the interventions on these subgroups are effective. Additionally, the various incidences of neutropenia, thrombocytopenia, and anemia cannot be explained at the HSC level. The highest incidence of neutropenia may be explained by the shortest life of neutrophils, making ANC the most sensitive reflection of hematotoxicity (132). Nevertheless, lineage-specific hematotoxic factors and lineage-differentiated vulnerability cannot be excluded as well.

Inflammation exerts a complex influence on HSCs including the direct interference and indirect impact *via* the regulation of niche factors. Chronic inflammation not only impairs the self-renewal and function of HSCs but also may increase their vulnerability or alter the response (59) to later inflammatory challenges. Whether acute inflammation will impair hematopoiesis in the long term during CAR-T therapy is an open question. Identifying the significant factors from all the candidates will be challenging. However, considering the clinical manifestations of CAR-T late hematotoxicity, the high incidence suggests that such factors may exist in most patients. The late event suggests that such factors may persist for a long time or exhibit late-phase peak levels long after the ablation of CRS or other earlier factors. Among these factors, cytokines, niche factors, and cellular immunity attacks, which are dominant to the pathogenesis of CAR-T late hematotoxicity, are crucial and need consideration. Identifying the most critical elements from all these factors will be challenging; however, the mechanisms are fundamental in understanding CAR-T late hematotoxicity.

## Author contributions

TS and XZ finished the conceptualization. TS performed document investigation, wrote the original draft, and prepared the table and figures. This work was supervised, revised, and edited by DL, LH, and XZ. All authors agree to be accountable for the content of the work. All authors contributed to the article and approved the submitted version.
